# Cauliflower Mosaic Virus TAV, a Plant Virus Protein That Functions like Ribonuclease H1 and is Cytotoxic to Glioma Cells

**DOI:** 10.1155/2020/7465242

**Published:** 2020-03-16

**Authors:** Valentina Turri, Olga S. Latinovic, Massimiliano Bonafè, Ngeh Toyang, Maria Parigi, Matteo Calassanzio, Pier Luigi Martelli, Alessandro Vagheggini, Giulia Abbati, Anna Sarnelli, Rita Casadio, Claudio Ratti, Paola Massi, James E. Schoelz, Maria S. Salvato, Filippo Piccinini, Giovanni Martinelli

**Affiliations:** ^1^Healthcare Direction, Istituto Scientifico Romagnolo per lo Studio e la Cura dei Tumori (IRST) IRCCS, 47014 Meldola, FC, Italy; ^2^Institute of Human Virology (IHV), University of Maryland, School of Medicine, 21201 Baltimore, MD, USA; ^3^Department of Microbiology and Immunology, University of Maryland, School of Medicine, 21201 Baltimore, MD, USA; ^4^Department of Experimental, Diagnostic and Specialty Medicine, University of Bologna, 40126 Bologna, Italy; ^5^Educational and Scientific (LLC), 21201 Baltimore, MD, USA; ^6^Istituto Zooprofilattico Sperimentale della Lombardia e dell'Emilia Romagna (IZSLER), 47122 Forlì, Italy; ^7^Istituto Oncologico Romagnolo (IOR), 47122 Forlì, Italy; ^8^Department of Agricultural and Food Sciences, School of Agriculture and Veterinary Medicine, University of Bologna, 40127 Bologna, Italy; ^9^Department of Pharmacy and Biotechnology, Interdepartmental Centre “L. Galvani” for Integrated Studies of Bioinformatics, Biophysics and Biocomplexity, Biocomputing Group, University of Bologna, 40126 Bologna, Italy; ^10^Unit of Biostatistics and Clinical Trials, Istituto Scientifico Romagnolo per lo Studio e la Cura dei Tumori (IRST) IRCCS, 47014 Meldola, FC, Italy; ^11^Biosciences Laboratory, Istituto Scientifico Romagnolo per lo Studio e la Cura dei Tumori (IRST) IRCCS, 47014 Meldola, FC, Italy; ^12^Medical Physics Unit, Istituto Scientifico Romagnolo per lo Studio e la Cura dei Tumori (IRST) IRCCS, 47014 Meldola, FC, Italy; ^13^Division of Plant Sciences, University of Missouri, 65211 Columbia, MO, USA; ^14^Scientific Directorate, Istituto Scientifico Romagnolo per lo Studio e la Cura dei Tumori (IRST) IRCCS, 47014 Meldola, FC, Italy

## Abstract

Recent comparisons between plant and animal viruses reveal many common principles that underlie how all viruses express their genetic material, amplify their genomes, and link virion assembly with replication. Cauliflower mosaic virus (CaMV) is not infectious for human beings. Here, we show that CaMV transactivator/viroplasmin protein (TAV) shares sequence similarity with and behaves like the human ribonuclease H1 (RNase H1) in reducing DNA/RNA hybrids detected with S9.6 antibody in HEK293T cells. We showed that TAV is clearly expressed in the cytosol and in the nuclei of transiently transfected human cells, similar to its distribution in plants. TAV also showed remarkable cytotoxic effects in U251 human glioma cells in vitro. These characteristics pave the way for future analysis on the use of the plant virus protein TAV, as an alternative to human RNAse H1 during gene therapy in human cells.

## 1. Introduction

Cancer remains a major public health problem worldwide. Current standards of cancer therapy include resection surgery (if applicable), radiation, chemotherapy, immunotherapy, and/or biological therapy [1]. The development of new therapies, such as targeted gene therapies, may provide an effective and nontoxic method of treating cancer.

A connection between the regression of cancer and viruses has long been theorized and reports of regression (cervical cancer, Burkitt's lymphoma, and Hodgkin's disease) after vaccination or infection with a related virus appeared in the early 20th century. Efforts to treat cancer through vaccination or infection with a virus deliberately started in the mid-20th century.

Plant viruses and vertebrate viruses are believed to exist in two different and nonoverlapping biological niches [2]. To date, plant viruses have not been described as pathogens for vertebrates or humans or known even to infect them [3]. Striking similarities have been observed between a plant pararetrovirus, Cauliflower mosaic virus (CaMV), and animal retroviruses, specifically their replication through productive reverse transcription and their high recombination rate [4]. However, there are also remarkable differences that include the tropism for two different kingdoms, the nature of the CaMV genome (a molecule of circular double-stranded DNA) and its lack of integration into host cell chromosomes [5]. Precisely, for its replication, CaMV is not dependent on the host DNA replication apparatus, in contrast to the geminiviruses (ssDNA plant viruses) that must overcome the lack of DNA replication factors in G_0_ cells, similar to the animal DNA tumor viruses such as SV40 and adenovirus [6]. There are just a few examples of plant viruses or plant virus proteins known to interact with human cells. Tobacco mosaic virus has been shown to induce endoplasmic reticulum stress-related autophagy in HeLa cells [7]. Tomato bushy stunt virus P19 has been shown to suppress RNA silencing in animal cells downstream of miRNA maturation [8, 9]. Although the CaMV 35S plant promoter is reported to be active in human enterocyte like cells [10], the expression of CaMV proteins in human cells has not been investigated.

Cauliflower mosaic virus (CaMV) is a DNA plant virus and is the type member of the family Caulimoviridae, which, together with the hepadnaviruses, belong to the group of pararetrovirus, which have a DNA genome but replicate by reverse transcription. The CaMV genome is a circular double-stranded DNA of approximately 8,000 bp and is encapsidated within a 50 nm icosahedral particle. CaMV infects principally plants of the Brassicaceae and Solanaceae families (turnips, cauliflowers, sprouts, and cabbages). Plant viruses replicate within an infected cell, move from cell to cell, and are transported from plant to plant. Once introduced within a host cell, virions migrate to the nuclear envelope, where they decapsidate. The viral genomes then enter the nucleus where all the gaps present in the genome are sealed and the covalently closed DNA then associates with host histones to form a supercoiled minichromosome that does not integrate into the host chromosomes and that is transcribed by the host RNA polymerase II to generate two mRNAs, the polycistronic 35S RNA comprising the entire genome encoding six proteins, and the 19S RNA encoding a single protein, the transactivator/viroplasmin protein (TAV), and the two mRNAs then move to the cytoplasm. In the cytoplasm, TAV is translated from the 19S RNA and aggregates in small inclusion bodies, where it transactivates translation of all other viral proteins from the 35S RNA [11]. TAV is recognized as a multifunctional effector interacting with a broad array of host proteins and either initiates the innate immunity reaction in a nonpermissive host or interferes with it in a permissive host [12–14]. TAV is composed of 520 amino acids that form an alpha-helical motif and is the least conserved protein within the CaMV genome. It contains a nuclear export signal (NES) at the N-terminus, a TAV domain, two nuclear localization signals (NLS), two RNA binding domains, and a putative zinc finger at the C-terminus [15]. In CaMV-infected plants, TAV is a nucleocytoplasmic shuttling protein [16] and nuclear import of TAV by the bipartite NLS and the nonconventional NLSa is likely through the importin alpha pathway. A minor part of TAV import could also occur through interactions between the TAV domain and L13 and L18 and other ribosomal proteins. This process causes TAV retention within the nucleolar, as opposed to the nucleoplasmic compartment [17]. TAV is a translational reinitiation factor that associates with the host translational machinery. This function is mediated by physical interactions between the TAV domain, the initiation factor eIF3 8 (subunit g), L13, L18, and L24 (ribosomal proteins) [16]. The hallmark of the RNase H/caulimovirus nucleic acid binding motif is a stretch of 40 amino acids with 11 highly conserved residues, seven of which are aromatic. Point mutations, insertions, and deletions indicated that the integrity of the motif is important for binding. The similarity between the RNase H and the caulimovirus domain suggests a common interaction with duplex RNAs of these two different groups of proteins [18]. However, little is known about TAV effects in the nonpermissive human host, and the aim of this study is to determine if plant virus proteins may have the same activity in mammalian and plant cells.

## 2. Materials and Methods

### 2.1. Computational Methods: Protein-DNA and Protein-Protein Interactions

Interactions between DNA and proteins from CaMV were predicted with DP-bind [19], a freely available program that combines three different tools (support vector machines, kernel logistic regression, and penalized logistic regression) to address the two-class classification problem consisting in recognizing DNA-binding and nonbinding residues.

Prediction considers the input sequence and the Position Specific Scoring Matrix (PSSM) compiled upon PSI-BLAST search for similar sequences. Consensus between two out of the three methods (majority consensus) has been adopted as a prediction criterion. The performance reported for the majority consensus method is 76% accuracy, 76% sensitivity, and 75% specificity [19]. Possible interactions between viral and human proteins have been inferred by screening the human proteome for sequences sharing similarity with known plant interactors and by analyzing the retrieved proteins in the context of the STRING human interactome [20]. Sequence similarity was searched with BLAST.

### 2.2. Computational Methods: Modelling of CaMV TAV Protein

The TAV three-dimensional structure is unknown and no template is available for the full-length modelling of the protein. To search for possible structural templates, the CaMV TAV sequence has been launched against Pfam [21], a database containing hidden Markov models for 17929 different protein domains. A 48-residue long domain significantly aligns with Pfam PF01693 (Cauli VI), which includes domains endowed with a three-dimensional structure in the protein database (PDB). Among them, the most similar to TAV's domain (target) is the hybrid domain of the human RNase H1, a specialized enzyme that can specifically resolve long DNA-RNA hybrids that can be used as a template [22]. In particular, we adopted the PDB file 3BSU [23], reporting the structure of the human domain in interaction with double-stranded RNA. The domain has been modelled with Phyre2 [24] on the basis of the hybrid domain of human RNase H1 (PDB code: 3BSU). The secondary structure for P6 protein has been predicted with PSIPRED [25].

### 2.3. Cell Culture and Transient Transfection

HEK293T cells (human embryonic kidney cell line 293 T cells that contain the SV40 T-antigen) were a gift of the European Brain Research Institute (EBRI) Rita Levi-Montalcini in Rome. HEK293T cells were grown in Dulbecco's modified Eagle's Medium High Glucose (DMEM High, Euroclone) supplemented with 10% fetal bovine serum (Euroclone) and 1% Penicillin/Streptomycin (Life Technologies) and they were cultured at 37°C and 5% CO_2_. A human glioblastoma cell line (U251, National Cancer Institute) was maintained in Eagle's Minimum Essential Medium (ATCC, 30-2003) supplemented with 10% Fetal Bovine Serum (FBS, Gibco, Thermo Fisher Scientific) at 37°C in a humidified 5% CO_2_ atmosphere. CaMV strain CM1841 full-length ORF I, III, IV, V, and VI (corresponding to MP, VAP, CP, EP, and TAV, respectively) were cloned from vector pS10-08 (CaMV strain CM1841 cloned into pBR322) into a mammalian expression vector pcDNA 3.1 (+) (EV), according to the native virus gene sequence (see database Genbank: V00140.1). The full-length ORF VI gene differs from the native sequence by a point mutation A402C (see database Genbank: V00140.1). pcDNA 3.1_P2A EGFP (EGFP) was used to test transient-transfection efficiency. HEK293T cells were seeded on coverslips within the wells of a 6-well plate at a density of 2 × 10^5^ cells/well (for confocal experiments) or in a T75 flask at a density of 3 × 10^6^ cells/flask (for western blot); cells were transiently transfected with 0.5 *μ*g of DNA/well and 8 *μ*g of DNA/flask of each DNA plasmid pcDNA3.1 (EV), pcDNA 3.1 ORF IV (CP), and pcDNA 3.1 ORF VI (TAV) (Genscript) with Polyethylenimine (PEI) 1 : 6 ratio with DNA. Experimental conditions include HEK293T cells transiently transfected with pcDNA 3.1 ORF IV (CP) and pcDNA 3.1 ORF VI (TAV), while controls include HEK293T cells not transfected and transiently transfected with EV and EGFP.

Transfection efficiency was found to be around 90% at 48 hours after transfection. U251 cells were seeded into a 24-well plate at a density of 2 × 10^4^ cells/well; cells were transiently transfected with 0.5 *μ*g of DNA/well of each DNA plasmid with 50 *μ*l of U251 Cell Avalanche transfection reagent mix (EZ Biosystems) 1 : 5 ratio with DNA. Experimental conditions include U251 cells transiently transfected with pcDNA 3.1 (+) ORF I (MP), pcDNA 3.1 ORF III (VAP), pcDNA 3.1 ORF IV (CP), pcDNA 3.1 ORF V (EP), pcDNA 3.1 ORF VI (TAV), and pcDNA 3.1 ORF VI in which the pathogenicity/host-range/avirulence domain of TAV12 (amino acids 2-113) was deleted (TAV deleted); negative controls include U251 cells not transfected, treated with transfection reagent only (media), and transiently transfected with EV and EGFP. Transfection efficiency was tested around 30–50% at 48 hours after transfection.

### 2.4. Cellular Fractionation

Cell's nucleus and cytoplasm separation was performed as previously described [26], with minor modifications. Nontransfected HEK293T cells and transiently transfected HEK293T cells (EV and TAV), 48 h after transfection, were twice washed in PBS 1X, resuspended in TM5 buffer (10 mM Tris, 5 mM MgCl2), and incubated 1 minute at room temperature and 5 minutes on ice during rotation. Triton X-100 (10% in H_2_O) was added and the suspension was passed through a syringe with a 22G needle and centrifuged for 10 minutes at 4°C. The supernatant (cytoplasm proteins) was collected and supplemented with Halt Protease and Phosphatase Inhibitor; then proteins were quantified using the Coomassie (Bradford) Protein Assay Kit (Thermo Fisher Scientific) and prepared for western blotting. Western blotting of cytosolic proteins was performed using histone H3 (Merck Millipore) for an endogenous nuclear marker, rabbit anti-TAV, and GAPDH-HRP-conjugated antibody (Origene, 2D9) as a control to show the absence of cytoplasm contamination. The pellet was resuspended in TM5 buffer, centrifuged twice for 10 minutes at 4°C, and the supernatant was aspirated to dry the nuclear pellet. Nuclear proteins were extracted using RIPA Buffer (150 mM NaCl, 1% Triton X-100, 0.5% sodium deoxycholate, 0.1% SDS, 50 mM Tris pH 8.0) supplemented with Halt Protease and Phosphatase Inhibitor and DNase 1 *μ*g/ml (Qiagen), incubated 30 minutes on ice in agitation, and then spun at 17000*g* for 15 minutes at 4°C. Nuclear proteins (supernatant) were collected and prepared for western blotting that was performed using the GAPDH-HRP-conjugated antibody (Origene, 2D9) for endogenous cytoplasm marker, anti-TAV rabbit polyclonal serum [27, 28], and histone H3 as a control for nuclear contamination.

### 2.5. Western Blot

HEK293T cells were transiently transfected with EV, CP, and TAV; nontransfected cells were used as a negative control. After 48 h, cells were lysed in RIPA Buffer with Halt Protease and Phosphatase Inhibitor, incubated 15 minutes on ice, and then spun at 17,000*g* for 15 minutes at 4°C. The amount of total protein was quantified using the Coomassie (Bradford) Protein Assay Kit (Thermo Fisher Scientific). The supernatants of each sample were collected and mixed with Bolt LDS Sample Buffer and Bolt Sample Reducing Agent (Thermo Fisher Scientific).

Samples were boiled for 5 minutes, loaded on a Bolt 4–12% Bis-Tris Gel (Thermo Fisher Scientific), and transferred to a PVDF nitrocellulose membrane (Biorad). The blots were blocked in 0.1% Tween-20 and 5% nonfat milk (Biorad) in Tris Buffered saline (Biorad) for 1 h at room temperature, and then membranes were tested for different antibodies: anti-TAV rabbit polyclonal serum (1 : 250) [19, 20] and anti-CP rabbit polyclonal serum (1 : 250, DSMZ AS-0206) overnight at 4°C, followed by the rabbit IgG-heavy and light chain antibody HRP-conjugated (Bethyl). Detection was performed using Clarity Western ECL substrate (Biorad), according to the manufacturer's instructions, and images were acquired using a Chemidoc XRS System (Biorad).

### 2.6. Plant Material and Infection

*B. rapa* plants (var. “Nagaoka”) were grown in a greenhouse at 20 ± 2°C with a 16 h photoperiod. After 15 days of growth, the plants were infected with an infectious CaMV clone, CaMV strain CM1841 excised from the pS10-08 vector (full-length CaMV strain CM1841 cloned into pBR322) by digestion with SalI. Mechanical inoculation was performed using Paul buffer (phosphate buffer 0.05 M pH 7.0, 5 mM DIECA, 1 mM EDTA, and 5 mM sodium thioglycolate) and celite as an abrasive powder. The typical symptoms of curling and leaf mosaic appeared two weeks after inoculation. Thirty-five days after infection, samples from infected plants were collected for analyses at the ultrastructural level. Noninfected *B. rapa* plants, inoculated with Paul buffer alone, were used as negative controls.

### 2.7. Tissue Processing for Transmission Electron Microscopy (TEM)

Several portions of the infected and uninfected leaf tissue were sampled using an ultrathin blade in the presence of 5% glutaraldehyde in 0.1 M potassium phosphate buffer (pH 7.2). In the same buffer, the HEK293T EV and TAV cells, at 48 h after transfection, were resuspended after centrifugation at 1000*g* for 5 minutes. Glutaraldehyde fixation was improved at the pressure of 2 bar for 20 min before embedding with an Araldite/TAAB812 Resin kit and mounting on the grids as previously described [29].

### 2.8. Immunocytochemical Labelling

Sample sections were processed as previously described [29] on grids for electron microscopy, adding 7% uranyl acetate to the contrasting step. After drying with the filter paper, the grids were examined with a 100 kV PHILIPS CM10 electron microscope.

### 2.9. HEK293T Cells Staining for Confocal Microscopy Analysis

HEK293T cells were grown on coverslips (0.17 mm thickness round cover glass 18 mm, Warner Instruments) in 6-well plate, and 0.5 *μ*g of DNA (pcDNA3.1, pcDNA 3.1 ORF IV (CP), and pcDNA 3.1 ORF VI (TAV)) with PEI 1 : 6 ratio with DNA was used for each well. Cells were fixed 48 hours after transient transfection in 4% paraformaldehyde (PFA) (Image-IT, Thermo Fisher Scientific) for 15 minutes at room temperature (RT). Afterwards, samples were permeabilized and blocked with 0.3% Triton X-100 and 5% FBS in PBS for 1 hour at RT. Coverslips were incubated with primary antibodies for CP and TAV (1 : 500 in blocking solution) overnight at 4°C, followed by the appropriate secondary antibody goat anti-rabbit Alexa Fluor 594 (Life Technologies, 1 : 600 in blocking solution) for 1 h at RT.

Fluorescence images were acquired on a Carl Zeiss Axioskop 40 Microscope. The following commercially available antibodies were used in immunofluorescence: polyclonal rabbit anti-CaMV IgG (DSMZ, AS-0206) and goat anti-rabbit IgG (H + L) cross-adsorbed secondary antibody conjugated to Alexa Fluor 594 (red color; 1 : 600 in blocking solution). TAV antiserum is a noncommercial rabbit anti-TAV polyclonal serum [27, 28].

### 2.10. Confocal Image Acquisition and Analysis

Confocal images of cell-associated fluorescence were acquired using the Zeiss LSM 800 confocal system (Carl Zeiss Microscopy, Germany). Three laser lines, 405 nm (blue, for nuclei), 488 nm (green, S9.6), and 561 nm (red, TAV), were used in these imaging experiments. Blue, green, and red signals were separated by a quad DAPI/FITC/TRITC/Cy5 dichroic beam splitter and were further acquired using a Gasp detector (Carl Zeiss LSM 800, Germany). A Plan-Apochromat 63x/1.4 Oil DIC objective (Carl Zeiss LSM 800, Germany) was used to visualize multicolored, labelled cell samples. All the parameters used in confocal microscopy were consistent in each experiment, including the laser excitation power, detector, and offset gain. Software Zen Blue (Carl Zeiss Microscopy) was used to generate original images and to collect z-stacks in order to achieve better three-dimensional information about the spatial location of TAV as subcellular localization within the cells (1 *μ*m thickness of sample slices). Negative control samples (nontransfected and EV) were stained with the same conditions as described above. All the images were acquired under the same instrumental settings. To assure the quality of acquired images, we took measurements with the same size of optical sections in three channels (405/488/561 nm). Furthermore, signal/noise ratio was assured by averaging data for every single image acquired. The saturated signal was avoided by using the range. Software Zen Blue was used to measure cell-associated intensity of the green events in nontransfected cells, EV, CP, and TAV samples. Each sample was examined by analyzing a large area [30] or at least 10 different imaging fields. Total intensity of the sample was measured and averaged among all images per set, in order to assure the statistics.

### 2.11. Cytotoxicity Assay

Cytotoxicity assays using trypan blue were performed on U251 cells not transfected, treated with media only, and transiently transfected with EV, MP, VAP, CP, EP, TAV, and TAV-deleted vectors. The results come from five biological replicates (technical duplicate of the data for each biological experiment). Cell viability data was acquired 5 days after transient transfection (as described above) using the Trypan Blue Assay reagent with the aid of the Countess II automated cell counter (Thermo Fisher Scientific). Cells were cultured as described above with a change of media after 72 h. The sample sizes of the vitality data differ as not every experimental condition has been analyzed in each experiment. Results are summarized as robust descriptive indices (median and interquartile range) that are not influenced by extreme values.

### 2.12. Statistical Analyses

The null hypotheses of equality between medians versus the alternative hypothesis of inequality have been inferred by means of the nonparametric Mann–Whitney rank sum test; *α* = 0.05 significance level has been set.

## 3. Results

### 3.1. TAV Sequence Similarity with the Human RNase H1

**Figure 1:**
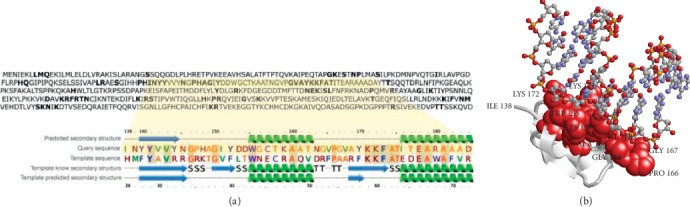
Modelling the 138-184 domain of CaMV TAV protein. (a) TAV sequence is shown, highlighting (in bold) residues putatively interacting with nucleic acids as predicted with DP-bind. Fragment 138-184 is aligned with the hybrid-binding domain of human RNase H1, whose structure is resolved (PDB: 3BSU) and has been adopted as a template. The alignment of structure-derived and computed secondary structures are also reported. (b) The structure of the double-stranded RNA molecule cocrystallized with the human protein is transferred on the modelled domain upon target-template superimposition. Balls and sticks representation is adopted for the RNA molecule. Residues represented with red Van Der Waals spheres are less than 0.5 nm distant from the RNA molecule. Residue marked ILE 138 is the N-terminus of the modelled domain.

We screened the human proteome for sequences sharing significant similarity with CaMV viral proteins, in order to elucidate the possible effects of viral proteins within human cells. Among CaMV proteins analyzed, TAV is the only one sharing significant similarity with a human protein (see [Supplementary-material supplementary-material-1] in the Supplementary Material for comprehensive analysis). TAV (UniprotKB: P03558) is a transactivator/viroplasmin protein (P6) involved in the translation of polycistronic viral DNA and its domains have been previously described [14, 31, 32].  Figure 1(a) shows the sequence alignment between target (TAV) and the hybrid domain of the human RNase H that we used as template (Protein Data Bank code: 3BSU) and the comparison between their secondary structures as predicted with PSIPRED (for both target and template) or derived from the structure (for the template). Out of 48 residues, 15 are conserved (31.3% sequence identity) [18]. From the structural alignment between template and model (that superimpose with a Root Mean Square Deviation of 0.15 nm), it is possible to infer possible RNA interaction sites for TAV.  Figure 1(b) shows the residues in the model with a distance lower than 0.5 nm from the RNA molecule transferred from the template. The hypothesis that the TAV domain can interact with human nucleic acids is corroborated by the fact that 15.2% of TAV residues are predicted to be protein-DNA interaction sites using the program DP-bind (Figure 1(a)). See [Supplementary-material supplementary-material-1] in the Supplementary Material for comprehensive analysis. We further characterized the role of TAV by searching for possible physical interactions of CaMV viral proteins with human proteins. The possible interactors of TAV in the natural host cells, in plants, have been previously reported [14, 33–37]. A clear similarity with human proteins is shown for some of the plant interactors, suggesting that human analogous can conserve the interaction with TAV. See [Supplementary-material supplementary-material-1] in the Supplementary Material for comprehensive analysis.

### 3.2. Nuclear Expression of TAV in Transiently Transfected HEK293T Cells

**Figure 2:**
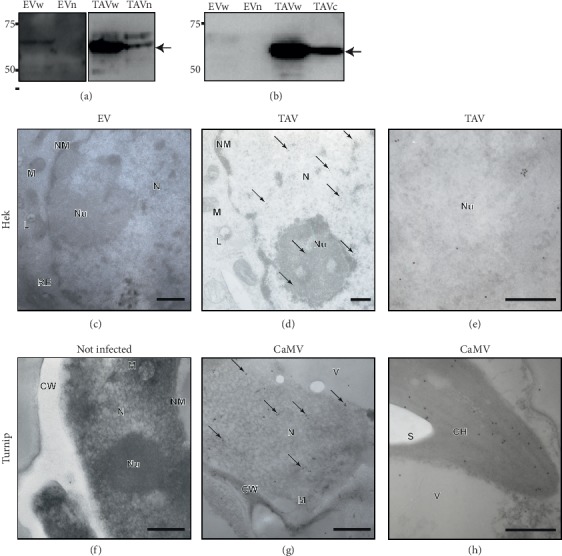
TAV detection with western blot (WB), transmission electron microscopy (TEM), and confocal microscopy. (a) Western blot shows total proteins (w) and nuclear proteins (n) of EV and TAV samples, immunoblotted with anti-TAV polyclonal serum. Arrow indicates TAV band (62 kDa), present in TAV sample only, in both W and N. Since the TAV antibody is a rabbit polyclonal antisera, it showed also a nonspecific band, also in EV sample just above the TAV band. Full-length blots/gels are presented in Supplementary [Supplementary-material supplementary-material-1]. (b) Western blot shows total proteins (w) and cytosolic proteins (c) of each sample, immunoblotted with anti-TAV polyclonal serum. Arrow indicates the TAV band, present in the TAV sample only. Full-length blots/gels are presented in Supplementary [Supplementary-material supplementary-material-1]. (c–h) Immunogold electron microscopy showing localization of CaMV TAV in Hek293T (c, d, e) and turnip cells (f, g, h). Ultrathin sections were immunostained with anti-TAV and gold-labelled secondary antibody. EV (c) and noninfected turnip cells (f) were used as a negative reference. Gold particles are indicated by arrows at low magnification (d, g) and clearly visible at higher magnification (e, h). The bars represent 200 nm. The letter symbols stand for the following: N: nucleus; Nu: nucleolus; NM: nuclear membrane; M: mitochondria; L: lysosome; RE: endoplasmic reticulum; CW: cell wall; CH: chloroplast; V: vacuole; S: starch.

In plants, TAV is actively transported into the nucleus through its two importin-α-dependent nuclear localization signals [17]. Since TAV is known to be a nucleocytoplasmic shuttle protein [16], we examined the nuclear expression of TAV in transiently transfected HEK293T cells (TAV-HEK293T cells), which are characterized by a high transfection efficiency and a high level of DNA/RNA hybrids, by using a nuclear separation protocol. Using western blot, we found that TAV is expressed exclusively in TAV-HEK293T cells, both in the cytoplasm and in the nucleus (Figures 2(a) and 2(b)). See Figures [Supplementary-material supplementary-material-1]–[Supplementary-material supplementary-material-1] in the Supplementary Material for comprehensive image analysis (the quality of the nuclear separation protocol is shown).

In order to confirm the nuclear expression of TAV, we analyzed EV and TAV- HEK293T cells using transmission electron microscopy (TEM). Only rare gold particles are seen randomly scattered over sections from healthy plants or EV- HEK293T cells indicating the absence of any endogenous substrates for the antibodies used (Figures 2(c) and 2(f)). Specific nuclear localization of gold particles was observed using the TAV antibody for TAV-HEK293T cells or CaMV-infected plant tissue labelling, respectively. TAV was localized intranuclearly both in TAV- HEK293T cells (Figures 2(d) and 2(e)) and in CaMV CM1841-infected turnip leaf cells which showed the typical symptoms of curling and leaf mosaic, used as a positive control (Figures 2(g) and 2(h)). Interestingly, nucleolar gold labelling on TAV-HEK293T samples (Figure 2(d)) and specific labelling of infected chloroplasts (Figure 2(h)) were observed using the antibody against TAV.

### 3.3. DNA/RNA Hybrids Reduction in TAV-HEK293T Cells

**Figure 3:**
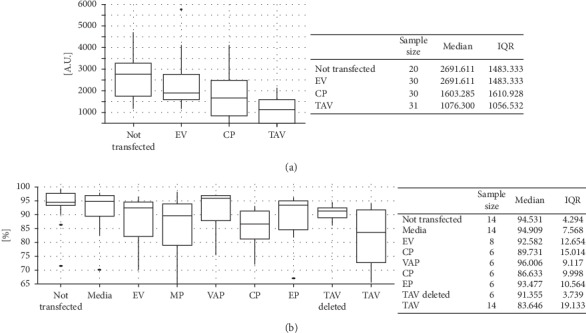
Quantification of TAV effects in human cells. (a) TAV-induced reduction of DNA/RNA hybrids in HEK293T cells. Boxplots and descriptive statistics of the fluorescence signal data in HEK293T cells. The interquartile ranges show that the cell-associated fluorescence signal values of EV and TAV are well concentrated around their median values, whereas nontransfected and CP values show higher dispersion. A high-fluorescence outlier has been identified for the EV control. (b) TAV-induced cytotoxic effects in U251 cells. Boxplots and descriptive statistics of the cytotoxicity data in U251 cells. Each box shows the first and third quartiles (bottom and top horizontal lines, respectively: box height is the interquartile range, IQR) and the median value (thick horizontal line) and whiskers' (vertical lines) ends are the lowest/highest datum within 1.5 × IQR from the box extremities; all values outside these intervals are considered as outliers (black dots). Tables collect sample size, median, and the interquartile range (IQR, i.e., the difference between the third and first quartiles).

Since TAV protein shows a homology with the mammalian RNase H1, we tested for its effect in reducing DNA/RNA hybrids in TAV-HEK293T cells, using an anti-R-loop antibody, S9.6 Ab [38]. As shown in the boxplots (Figure 3(a)), the S9.6 Ab fluorescence signal registered in TAV-HEK293T cells had a median value of 1,076 arbitrary units (AU, i.e., grey levels at 16-bit resolution) which is lower when compared to 2,692, 1,938, and 1,603 AU for nontransfected cells, EV, and CP-HEK293T cells, respectively. Empirical evidence leads one to firmly reject the null hypothesis when comparing TAV-HEK293Tcells with EV-HEK293T cells (*P* value <0.001); indeed, both graphical and descriptive comparisons point out substantial differences. On the contrary, the difference between the central tendency of CP and EV-HEK293T cells was not statistically significant (*P* value = 0.061). Taken together, our results suggest that TAV acts like RNase H1 protein in human cells, while the median lower level of CP is probably to be ascribed to its close association with other nucleic acid binding proteins or to the nucleic acid binding of CP through its own predicted motifs (e.g., the zinc finger motif, CXCX_7_HX_14_H, described in the PROSITE entry PDOC50158 [39]). See [Supplementary-material supplementary-material-1] in the Supplementary Material for comprehensive analysis.

### 3.4. TAV's Cytotoxicity to Human Glioma Cells (U251 Cells)

We then sought to investigate whether TAV interferes with cell viability. To this aim, cytotoxicity assays using trypan blue were performed on U251 human glioma cells, which have been transiently transfected with the CaMV proteins analyzed. As shown in the boxplots (Figure 3(b)), the vitality data of TAV-U251 samples reveal the lowest median values. This is more noticeable for the negative controls (83.6% for TAV compared to around 94.5%, 94.9%, and 92.6% for nontransfected cells, media only, and EV, respectively) and VAP and EP (96.0% and 93.5% median vitality, respectively). TAV-deleted median vitality data is 91.4%, therefore comparable to negative controls. Movement protein (MP) and CP median vitality values (89.7% and 86.6%, respectively) are closer to the median TAV one. The interquartile ranges show that the vitality values of nontransfected and TAV-deleted are highly concentrated around their median values, whereas MP and TAV show higher dispersion. Empirical evidence leads one to firmly reject the null hypothesis when comparing TAV with nontransfected (*P* value <0.001) and media-control values (*P* value = 0.004); indeed, both graphical and descriptive comparisons point out substantial differences.

## 4. Discussion

Our results reveal a new and unexpected role for CaMV TAV in reducing DNA/RNA hybrids in HEK293T cells and being cytotoxic for U251 cells. DNA/RNA hybrids are formed continuously throughout the genome during RNA transcription and DNA duplication [40]. Together with the displaced single-strand DNA filament, DNA/RNA hybrids are collectively called R-loops [40]. R-loops are a threat to genome integrity due to their capability to promote DNA double-strand breaks, chromosome rearrangements, and replication fork stalling [40]. R-loops behave as hotspots of genomic instability in a variety of organisms. Current models suggest that uncontrolled R-loops are a hazard to genome integrity, and the expression of RNA-DNA hybrid-binding proteins in various cancer types is associated with survival [41]. Accordingly, mechanisms that prevent R-loop accumulation (e.g., THO/TREX and BRCA2 [39, 40]) enhance genome stability in human cells [39], whereas the sequestration of hTREX by Kaposi's sarcoma-associated herpesvirus protein ORF 57 leads to R-loop formation and genome instability [40]. Nevertheless, DNA/RNA hybrids exert important physiologic roles in gene regulation, as they control gene expression activation and termination [40]. Moreover, depletion of DNA/RNA hybrids by RNase H1 overexpression has been shown to impair telomere homeostasis in cancer cells that maintain telomere length via the telomerase-independent “alternative lengthening of telomeres” (ALT) pathway [42]. Indeed, these tumor cells need to maintain precise levels of DNA/RNA hybrids to support telomeric homologous recombination (HR) without compromising telomere integrity. Together with our findings, these data suggest that particular sets of cancer cells, such as those carrying active ALT pathway, may be preferentially sensitive to DNA/RNA hybrid depletion and may be suitable targets for TAV/RNase H1 overexpression-based gene therapy.

## 5. Conclusions

These proof-of-concept data open the door to cross-kingdom use of plant virus proteins in human therapies. Since RNase H-mediated degradation of ASOs, which form an RNA/DNA hybrid once bound to the RNA, is now under clinical investigation for amyotrophic lateral sclerosis among other neurological diseases [43], TAV—a plant virus protein—may become a key player in future gene therapy scenarios in a variety of human diseases, including cancer and neurodegeneration.

## Data Availability

All data used in the analysis is available in the main text or the supplementary materials.

## Supplementary Materials

Supplementary Materials Supplementary Table 1: characterization of CaMV proteins and their putative interactions with human proteins and nucleic acids. Together with Figure 1(a) and Supplementary Table 3, these data give a whole overview of possible human proteins interactors with CaMV proteins. Supplementary Table 2: RNA interacting sites in TAV inferred from the modelling procedure. These data complete the model presented in Figure 1(b). Supplementary Table 3: proteins interacting with CaMV TAV in plants and corresponding proteins in humans. Supplementary Figure 1: full-length blot/gel related to Figure 2(a). Supplementary Figure 2: full-length blot/gel related to Figure 2(b). Supplementary Figure 3: western blot detection of GAPDH and Histone H3 as cytoplasmic/nuclear separation controls. Supplementary Figure 4: residues putatively involved in binding DNA and RNA have been predicted with the machine-learning method DP-bind and are presented in this figure.
