# Supplementary material for: Cauliflower Mosaic Virus TAV, a Plant Virus Protein That Functions like Ribonuclease H1 and is Cytotoxic to Glioma Cells
Source: Biomed Res Int. 2020 Mar 16;2020:7465242. doi: 10.1155/2020/7465242 (PMC7102451; doi:10.1155/2020/7465242)
Supplement: Supplementary Materials — Supplementary Table 1: characterization of CaMV proteins and their putative interactions with human proteins and nucleic acids. Together with Figure 1(a) and Supplementary Table 3, these data give a whole overview of possible human proteins interactors with CaMV proteins. Supplementary Table 2: RNA interacting sites in TAV inferred from the modelling procedure. These data complete the model presented in Figure 1(b). Supplementary Table 3: proteins interacting with CaMV TAV in plants and corresponding proteins in humans. Supplementary Figure 1: full-length blot/gel related to Figure 2(a). Supplementary Figure 2: full-length blot/gel related to Figure 2(b). Supplementary Figure 3: western blot detection of GAPDH and Histone H3 as cytoplasmic/nuclear separation controls. Supplementary Figure 4: residues putatively involved in binding DNA and RNA have been predicted with the machine-learning method DP-bind and are presented in this figure. [file 7465242.f1.pdf]

Supplementary Materials

Supplementary Table 1: Characterization of CaMV proteins and of their putative interactions with human proteins and nucleic acids. Together with Figure Fig. 1a and Supplementary Table 3, these data give a whole overview of possible human proteins interactors with CaMV proteins.

Supplementary Table 2: RNA interacting sites in TAV inferred from the modeling procedure. These data complete the model presented in Figure 1b.

Supplementary Table 3: Proteins interacting with CaMV TAV in plants and corresponding proteins in human.

Supplementary Figure 1: Full length blot/gel related to Fig. 2a.

Supplementary Figure 2: Full length blot/gel related to Fig. 2b

Supplementary Figure 3: Western blot detection of GAPDH and Histone H3 as cytoplasmic/nuclear separation controls.

Supplementary Figure 4: Residues putatively involved in binding DNA and RNA have been predicted with the machine-learning method DP-Bind and are presented in this figure.

	UniprotKB in *CaMV*	Functional features in plants	Structural features	Predicted DNA and RNA binding sites	Similar human protein	Possible human protein interactors
P1	P03546	Movement protein (MP) Transport of virus in host, cell to cell	Coiled-coil domains<br>Homotrimer<br>No PDB<br>Interacts with plant Prenylated Rab acceptor protein 1D	Yes	None	Prenylated Rab acceptor protein 1 (by similarity with plant protein) and its 30 interactors, including syntaxins and members of RAS family (from STRING database)
P3	P03551	Virion-associated protein (VAP) DNA-binding	Coiled-coil domains<br>Homotetramer<br>PDB: 3F6N, 3K4t	Yes	None	None
P4	P03543	Capsid protein (CP) Viral penetration into host nucleus. Nuclear Localization Signal	Contains Zn-finger motif<br>Self-assemble into 420-mer capsid<br>No PDB	Yes	None	Importin-alpha (based on the presence of a Nuclear Localization Signal)
P5	P03555	Enzymatic polyprotein (EP): endopeptidase activity, endonuclease activity, reverse transcriptase acrivity	Reverse transcriptase and protease domains<br>No PDB	Yes	None	None
P6	P03558	Transactivator-viroplasmin (TAV)<br>Translation regulation<br>Nuclear localization signal	A domain can be modelled using RNase H1	Yes	RNase H1	See Supplementary Table 3

Table S1: Characterization of CaMV proteins and of their putative interactions with human proteins and nucleic acids. UniProtKB codes of CaMV proteins are reported. Structural and functional features are retrieved from UniProtKB entries. Possible interactions with nucleic acids are computed with DP-pred. Human proteins sharing similarity have been retrieved with a Blast search against UniProtKB setting a 10-3 threshold on the e-value. Human interactors are inferred on the basis of sequence similarity with viral proteins or with known plant interactors and are analysed in the context of the STRING human interactome (protein data bank, PDB).

3BSU (template)	TAV protein (target)	Conservation
TRP 43	TRP 155	Conserved
ARG 47	LYS 159	Similar
ARG 52	GLY 164	Non similar
PRO 54	PRO 166	Conserved
ALA 55	GLY 167	Similar
ALA 56	VAL 168	Similar
ARG 57	ALA 169	Non similar
PHE 58	TYR 170	Similar
LYS 59	LYS 171	Conserved
LYS 60	LYS 172	Conserved

Table S2: RNA interacting sites in TAV inferred from the modelling procedure. The 10 residues of TAV interacting with RNA are listed. 4 out of 10 residues are conserved between target and template, while 4 more are substituted with similar residues.

Plant protein	Human analogous	Seq. Id. % / Coverage %	KEGG Pathway (Human protein)
Elongation factor P-TEFb<br>CDKC1 (Q9LFT8)<br>CDKC1 (Q8W4P1)	CDK9 (P50750)<br>CDK9 (P50750)	47 / 64<br>47 / 63	Transcriptional mis-regulation in cancer
Initiation factors<br>eIF3g (F4J6A1)<br>eIF4g (Q76E23)<br>eIF2b (Q41969)	eIF3g (O75821)<br>eIF4g (O43432)<br>eIF2b (P20042)	37 / 90<br>28 / 35<br>64 / 58	RNA transport
Ribosomal proteins<br>L13 (Q9SYL9)<br>L18 (Q9SX68)<br>L24 (P92959)	L13 (P26373)<br>-<br>L24 (Q96A35)	35 / 46<br>-<br>25 / 48	Ribosome
Suppressor of apoptosis<br>Bax Inhibitor (Q9LD45)	TMBIM6 (P55061)	43 / 89	Non annotated
Dicer-like proteins<br>DCL4 (P84634)	DICER1 (Q9UPY3)	30 / 72	MicroRNAs in cancer
Chloroplast positioning<br>CHUP1 (Q9LI74)	*PPFIBP1 (Q86W92)*	*22 / 11*	Non annotated
Cytochrome b6-f complex<br>RISP (Q9ZR03)	UQCRFS1 (P47985)	56 / 19	Oxidative phosphorylation
Cys-rich secretory protein<br>PDLP1 (Q8GXV7)	*IQSEC3 (Q9UPP2)*	*63 / 6*	*Endocytosis*
SRC2 (O04023)	*GRINA (Q7Z429)*	*44 / 33*	Non annotated
Transcription factor<br>FIT (Q0V7X4)	*HEY2 (Q9UBP5)*	*30 / 30*	*Human papillomavirus infection*
Protein kinase<br>TOR (Q9FR53)<br>S6K1 (P42818)	mTOR (P42345)<br>RPS6KB1 (P23443)	37 / 97<br>48 / 68	mTOR signaling pathway
Regulatory proteins<br>NPR1 (P93002)<br>NPR3 (Q8L746)<br>NPR4 (Q5ICL9)	ANK2 (Q01484)<br>ABTB1 (Q969K4)<br>NACC2 (Q96BF6)	36 / 28<br>34 / 14<br>36 / 12	Proteoglycans in cancer
DRB4	E2AK2 (P19525)<br>ADAR (P55265)	28 / 52<br>28 / 38	Protein processing in endoplasmic reticulum/ Cytosolic DNA-sensing pathway

Table S3: Proteins interacting with CaMV TAV in plants and corresponding proteins in human. For CaMV TAV interactors in plants, in some cases there is a direct interaction between CaMV and the plant protein, whereas in others the interaction is inferred due to an effect on function. Proteins in bold are highly significant matches (e-value < 10-10). Proteins in italics are not significant matches (e-value > 0.001). For each interactor, the most similar human proteins are reported along with the sequence identity and the coverage.

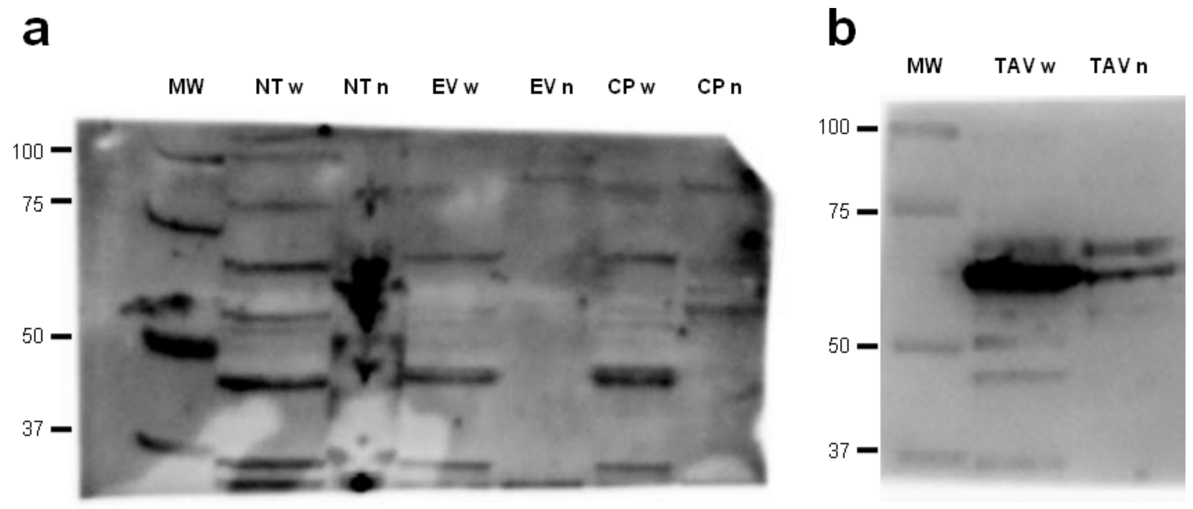

Figure S1: Full length blots/gels related to Fig. 2a. **(a)** Full length blot/gel from which 50-75 kDa EV whole (w) and EV nuclear (n) were cropped. Molecular weight (MW, marker) and negative controls cells (not trasfected cells, NT w and NT n, and cells transfected with capsid protein, CP w and CP n) have been omitted. **(b)** Full length blot/gel from which 50-75 kDa TAV w and TAV n were cropped. All samples were loaded on the same gel, but we cut the membrane in two (**a**, **b**) since we first immunoblotted the membrane **a** with anti-CP polyclonal serum and then with anti-TAV polyclonal serum (after stripping). Membrane **b** was tested with anti-TAV polyclonal serum only.

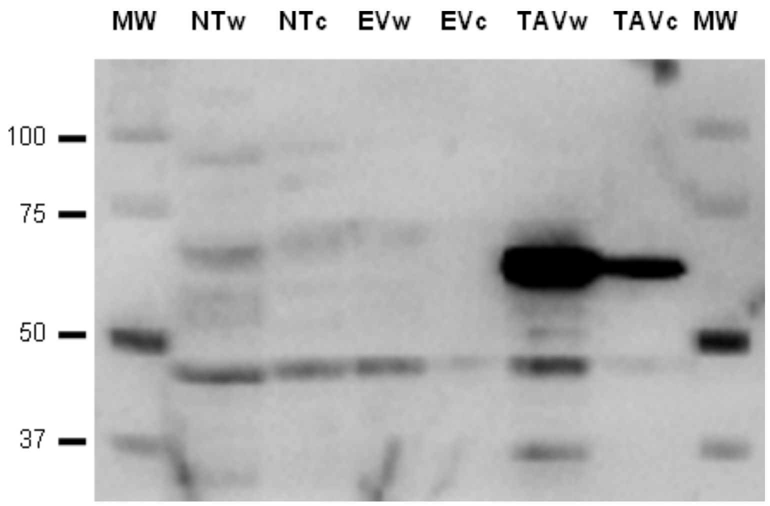

Figure S2: Full length blot/gel related to Fig. 2b. Full length blot/gel from which 50-75 kDa EV and TAV, both whole (w) and cytosolic (c), were cropped. Molecular weights (MW, marker) and not trasfected cells, NT w and NT c, have been omitted. This membrane has been immunoblotted with anti-TAV polyclonal serum only.

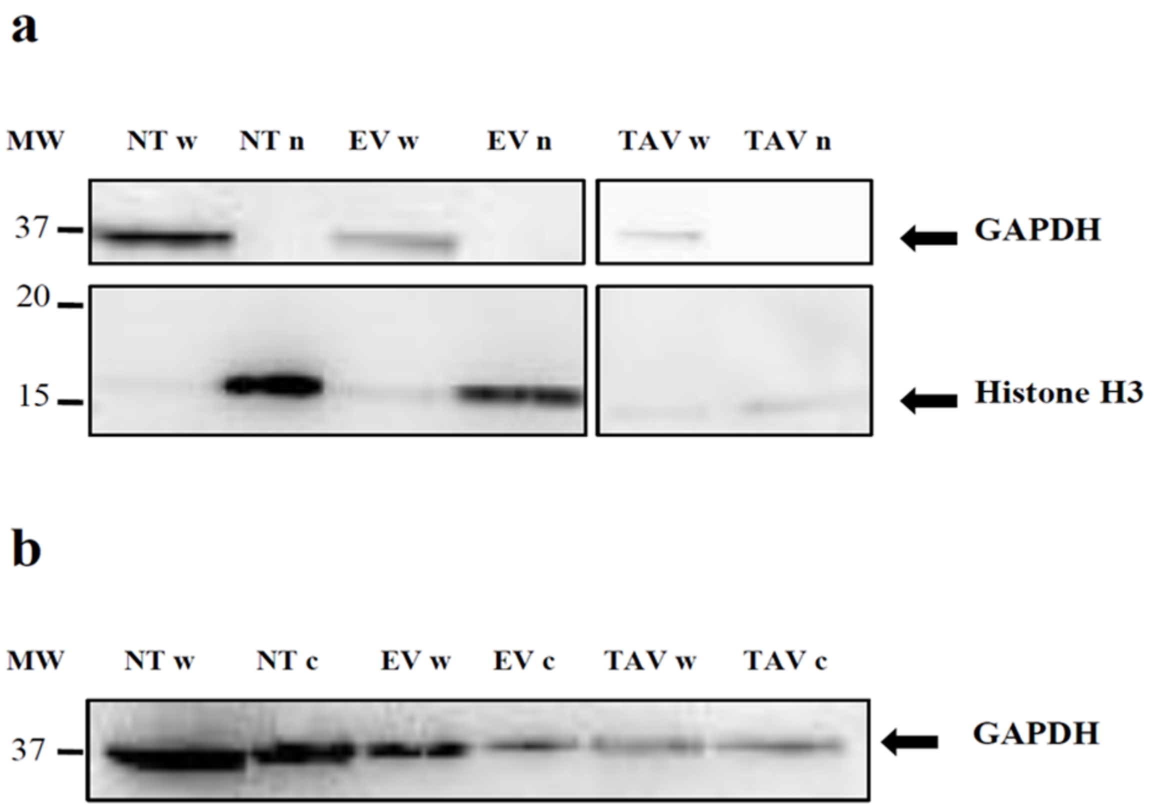

Figure S3: Western blot detection of GAPDH and Histone H3 as cytoplasmic/nuclear separation controls. (**a**) GADPH was used to check for contamination of nuclear fraction and a specific band for GAPDH (37 kDa, cytosolic) was detected in all the samples (not transfected - NT, EV and TAV HEK293T cells) but not in their nuclear fractions, as expected. On the contrary, histone H3 was used to check for contamination of cytosolic fraction and a specific band for histone H3 (17 kDa, nuclear) was detected just in their nuclear fraction, as expected; in TAV, the histone H3 band is weaker than others, but it is present. Arrows indicate the presence of GAPDH and histone H3. Full length blots/gels are presented in Supplementary Figure 4. (**b**) A specific band for GAPDH, used as cytosolic control, was present in all samples (NT, EV and TAV HEK293T cells) both whole and cytosolic, as expected; the band for histone H3 was never detected (data not shown) since that protein was weakly expressed compared to the total protein amount. The letter symbols stand for following: w, whole fraction; n, nuclear fraction; c, cytosolic fraction.

P1 984 base pairs (bp) - Movement protein (MP)

MDLYPEENTQSEQSQNSENNMQIFKSENSDGFSSDLMISNDQLKNISKTQLTLEKEKIFKMPNVLSQV
MKKAFSRKNEILYCVSTKELSVDIHDATGKVYLPLITREEINKRLSSLKPEVRKIMSMVHLGAVKILL
KAQFRNGIDTPIKIALIDDRINSRRDCLLGAAKGNLAYGKFMFTVYPKFGISLNTQRLNQTLSLIHDFE
NKNLMNKGDKVMTITYIVGYALTNSHHSIDYQSNATIELEDVFQEIGNVQQSDFCTIQNDECNWAIDI
AQNKALLGAKTQSQIGNSLQIGNSASSSNTENELARVSQNIDLLKNKLKEICGE

P3 390 bp - Virion-associated protein (VAP)

MANLNQIQKEVSEILSDQKSMKSDIKAILELLGSQNPTKESLEAVAAKIVNDLTKLINDCPCNKEILEA
LGNQPKEQLIEQPKEKGKGLNLGKYTYPNYGVGNEELGSSGNPKALTWPFKAPAGWPNQF

P4 1467 bp - Capsid protein (CP)

MAESILDRTINRFWYNLGEDCLSESQFDLMIRLMEESLDGDQIIDLTSLPSDNLQVEQVMTTTDDSIS
EESEFLLAIGEISEDESDSGEEPEFEQVRMDRTGGTEIPKEEDGEGPSRYNERKRKTPEDRYFPTQPKTI
PGQKQTSMGMLNIDCQINRRTLIDDWAAEIGLIVKTNREDYLDPETILLLMEHKTSGIAKELIRNTRW
NRTTGDIIEQVINAMYTMFLGLNYSDNKVAEKIDEQEKAKIRMTKLQLFDICYLEEFTCDYEKNMYK
TEMADFPGYINQYLSKIPIIGEKALTRFRHEANGTSIYSLGFAAKIVKEELSKICDLSKKQKKLKKFNK
KCCSIGEASVEYGGKKTSKKKYHKRYKKRYKVYKPYKKKKKFRSGKYFKPKEKKGSKRKYCPKG
KKDCRCWICNIEGHYANECPNRQSSEKAHILQQAENLGLQPVEEPYEGVQEVFILEYKEEEEETSTEE
SDDESSTSEDSDSD

P5 2043 bp - Enzymatic polyprotein (EP)

MDHLLLKTQTQIEQVMNVTNPNSIYIKGRLYFKGYKKIELHCFVDTGASLCIASKFVIPEEHWVNAE
RPIMVKIADGSSITISKVCKDIDLIIAGEIFKIPTVYQQESGIDFIIGNNFCQLYEPFIQFTDRVIFTKNKSY
PVHITKLTRAVRVGIEGFLESMKKRSKTQQPEPVNISTNKIENPLEEIAILSEGRRLSEEKLFITQQRMQ
KIEELLEKVCSENPLDPNKTKQWMKASIKLSDPSKAIKVKPMKYSPMDREEFDKQIKELLDLKVIKP
SKSPHMAPAFLVNNEAEKRRGKKRMVVNYKAMNKATIGDAYNLPNKDELLTLIRGKKIFSSFDCKS
GFWQVLLDQESRPLTAFTCPQGHYEWNVVPFGLKQAPSIFQRHMDEAFRVFRKFCCVYVDDILVFS
NNEEDHLLHVAMILQKCNQHGIILSKKKAQLFKKKINFLGLEIDEGTHKPQGHILEHINKFPDTLEDK
KQLQRFLGILTYASDYIPKLAQIRKPLQAKLKENVPWKWTKEDTLYMQKVKKNLQGFPPLHHPLPE
EKLIIETDASDDYWGGMLKAIKINEGTNTELICRYASGSFKAAERNYHSNDKETLAVINTIKKFSIYLT
PVHFLIRTDNTHFKSFVNLNYKGDSKLGRNIRWQAWLSHYSFDVEHIKGTDNHFADFLSREFNKVNS

Figure S4: Residues putatively involved in binding DNA and RNA have been predicted with the machine-learning method DP-Bind[20]. Residues predicted to interact with nucleic acids are highlighted in yellow.
